# The relationship between childhood emotional abuse and borderline personality disorder: the mediating role of difficulties in emotion regulation among Lebanese adults

**DOI:** 10.1186/s40479-023-00241-0

**Published:** 2023-11-21

**Authors:** Gaelle Kanj, Souheil Hallit, Sahar Obeid

**Affiliations:** 1 School of Arts and Sciences, Holy Spirit University of Kaslik, P.O. Box 446, Jounieh, Lebanon; 2 School of Medicine and Medical Sciences, Holy Spirit University of Kaslik, P.O. Box 446, Jounieh, Lebanon; 3 Applied Science Research Center, Applied Science Private University, Amman, Jordan; 4 Research Department, Psychiatric Hospital of the Cross, Jal Eddib, Lebanon; 5 Social and Education Sciences Department, School of Arts and Sciences, Lebanese American University, Jbeil, Lebanon

**Keywords:** Borderline Personality Disorder, Difficulties in emotion regulation, Childhood emotional abuse, Lebanon

## Abstract

**Objective:**

The present study investigates the mediating effect of difficulties in emotion regulation in the association between childhood emotional abuse and Borderline Personality Disorder (BPD) among Lebanese adults.

**Method:**

This cross-sectional study, involving 411 participants, was conducted between March and August 2022. Lebanese individuals from all governorates of the country were recruited using the Snowball Sampling technique. Three self-report scales were utilized to complete this research; the ‘Difficulties in Emotion Regulation Scale—Brief Version (DERS-16)’ which evaluates the difficulties in emotion regulation of individuals, the ‘Childhood Trauma Questionnaire—Short Form (CTQ-SF)’ which grants a subjective evaluation of the general childhood environment of the participants, as well as the ‘Borderline Personality Questionnaire (BPQ)’ which measures Borderline Personality Disorder traits, that demonstrate significant convergence with the disorder.

**Results:**

The results indicate that DERS-16 played an indirect effect role between childhood emotional abuse scores and Borderline Personality Disorder. Higher emotional abuse scores were significantly associated with higher DERS-16 scores, which in turn was significantly associated with higher BPQ scores. Moreover, childhood emotional abuse was directly associated with higher BPQ scores.

**Conclusion:**

This work suggests that, among the different forms of childhood abuse, emotional abuse may have a role in the development of Borderline Personality Disorder. Training on emotion regulation strategies would potentially benefit individuals in preventing BPD development and facilitating therapeutic processes.

## Introduction

Borderline Personality Disorder (BPD) is characterized by a generalized pattern of instabilities concerning one’s identity and sense of self, interpersonal relationships as well as affects [1, 2]. The semiology of this disorder also includes episodes of frenzied anger, marked impulsivity, suicidal or self-harm behaviors, paranoid ideation related to stress or severe dissociative symptoms, chronic feelings of emptiness and frantic efforts to avoid abandonment, whether real or imagined [1]. With respect to emotional dysregulation, individuals with BPD display affective instability and often respond to stimuli with manifestations of intensely experienced and expressed dysphoric affects, such as anxiety, depression, or irritability [2, 3]. These negative emotional states and/or maladaptive outbursts of anger could lead to physical altercation with others or self-destructive conduct resulting from a weak capacity for impulse control [2, 3]. Estimates note a prevalence of 5.9% at the onset of this debilitating and severe psychiatric disorder among the general population, and of 6%, 10% and 20% respectively in primary care settings, outpatient mental health clinics and psychiatric hospitals [1, 2]. The diagnosis of Borderline Personality Disorder was seen in Egypt in 13.5% of outpatients suffering from anxiety, dissociative, somatoform or adjustment disorder, and in 3.8% adults in primary care in the United Arab Emirates [4]. No specific data concerning this disorder has been reported in Lebanon, to the extent of our knowledge [4], which motivated the inclusion of BPD as one of this research’s variables.

Furthermore, consistent with various developmental models’ suggestions, the pathology of Borderline Personality Disorder is shaped by a combination of biological and environmental mechanisms [5]. On that account, the multifactorial model of Zanarini and Frankenburg [6] considers as an etiological factor to BPD’s development, the traumatic environment within the household, which might be determined by a variety of experiences including prolonged early separation of the parents or child abuse [7, 8]. The latter, in its various forms such as sexual, physical, and emotional, is suggested to be central to the development of BPD [9]. In this regard, sexual abuse would increase the incidence of self-harm or suicidal behavior, and/or identity disturbance among other symptoms occurring in BPD semiology [10]. Difficulties in developing a self-identity and forming stable relationships with others might as well derive from experiences of early physical abuse [11–13]. Traumatic environment might also be described as invalidating; then translating a delegitimization of the emotional expression or experience of a child [14]. That invalidating environment is composed of four main characteristics as follows: (1) discouragement of negative emotional expression; (2) misattribution; (3) inaccuracy; and (4) oversimplification of problem solving [14]. In other words, in this type of environment, the expression of one’s emotions and needs would constantly be neglected, ignored, or even punished [15]. Consequently, these disabling experiences could shepherd the victim towards internalizing self-invalidating behaviors and failing to learn adequate emotion regulation (ER) strategies [15]. Along these lines, attempting to regulate negative emotions by resorting to inappropriate techniques such as self-harm behaviors, would characterize Borderline Personality Disorder [15]. In fact, according to a study conducted among 1028 Lebanese children aged between 8 and 17 [16], BPD is predicted by the family size and aspects of its functioning; communication and affect, as well as gender, age and parental education. In line with this same study, 65% and 54% of children suffered from at least one incident of psychological and physical violence, respectively, in one year. In addition, emotional, physical and sexual abuse and neglect have been reported in the literature as significant risk factors for BPD in adults [17, 18]. In this context, and relative to the results of a recent meta-analysis [9] as well as those of a Turkish study [4], experience of early trauma is delineated more frequently by individuals presenting Borderline Personality Disorder than by those suffering from other types of psychiatric disorders or by control groups. Similarly, in a sample of women with BPD, 75% point to a history of childhood sexual abuse, and more than 50% claim to have been abused before the age of six years [19].

Diverse forms of abuse usually befall simultaneously; sexual abuse often coincides with neglect, emotional abuse and/or physical abuse [7, 20, 21]. Subsequently, sexual abuse is unlikely to occur in the absence of emotional abuse, which is, nonetheless, the form of abuse that is most probable to betide independently of others [22–24]. The latter would uniquely presage the development of BPD; incidents of emotional abuse such as degradation and/or disregard, experienced during one’s childhood, would be more intensely linked to emotional dysregulation in adulthood than that of sexual or physical abuse [5, 17, 25], increasing the interest in considering emotional abuse as one of the study’s variables. Indeed, children’s development of adequate emotion regulation skills and expression of appropriate affect would be jeopardized by experiences of emotional abuse [26, 27]. This could instill in the child the belief that their emotional expression or efforts at regulating these emotions are intolerable, leading to the adoption of poor ER strategies including emotional avoidance and/or inhibition, among others [26, 28, 29]. Because of incidents of early emotional abuse, “response-focused” aspects of ER would also show deficits, involving impulsive or aggressive reactions and enaction of inappropriate emotion regulation strategies in tough situations [26, 30, 31].

In this vein, the biosocial model of Linehan [32] deems BPD as the result of an incapacitating and unstable environment for social education, supplemented by a biological predisposition to difficulties in emotion regulation. Thus, in line with the preceding, child abuse appears to disrupt the normal process of emotional development, leading to a major manifestation or a hallmark feature of Borderline Personality Disorder; that is emotional dysregulation [33]. The latter, with a prevalence rate of 13.9% in the general British adult population [34] and an average of 27.17 ± 16.48 (mean ± standard deviation) among 258 participants of a cross-sectional study conducted in Lebanon [35], is on one hand, operationalized in terms of the emotional response’s characteristics; intense response or inability to recognize one’s emotions, and on the other hand, in terms of deficits in the capacity to use adapted strategies for regulating emotions, such that individuals with BPD would eventually turn to rumination or even to blaming others or oneself [17, 36]. In other words, emotional dysregulation, defined as a key factor in the development and maintenance of BPD, would refer to the inefficacy or difficulty in identifying, understanding, accepting, and processing emotions, controlling impulsive behaviors as well as modulating emotional responses [37, 38]. Fighting undesirable emotions would then occur inadequately through the implementation of inappropriate emotional regulation strategies, or the failure of adapted ones [39]. Difficulties in regulating emotions would, in that sense, highlight fluctuations in emotions, mood or affects, remarkable for their complex controllability, frequency, intensity and speed [40]. These difficulties would also imply an inability to refrain from impulsive conduct or to use effective emotional regulation strategies [37, 41].

In addition, substantial research indicates a connection between difficulties in emotion regulation and the development of BPD [5]. In that context, individuals with BPD often report experiencing challenges with regulating their emotions, a lessened willingness to tolerate distress in pursuit of their goals, and an avoidance of their daunting emotions. On that account, difficulties in emotion regulation could be considered as a core feature of BPD, and/or a main cause of the said disorder’s development, as it may also fail to be its primary factor and occur in other mental health conditions for which it represents a vulnerability factor [42–48].

Furthermore, researchers have identified several factors that may contribute to emotional dysregulation in individuals with Borderline Personality Disorder, which include a history of trauma or abuse, potentially leading to difficulties in regulating emotions and developing coping skills [49, 50]. In fact, emotionally insurmountable hurdles—by virtue of their violence and instability—endured by the child within the family home would require the establishment of useful tactics to combat them [51]. These, in turn, would replace and compromise the achievement of the child’s individual emotional objectives [51]. For instance, among a group of children aged between seven and ten years old, an association has been observed between child maltreatment and emotional lability, contributing to poorer outcomes in terms of emotion regulation [52]. On that account, under-regulation of affect was found to partially mediate the relationship between childhood trauma and the presence of BPD symptoms in adulthood [42]. An indirect connection between emotional abuse during childhood and the characteristics of BPD, through difficulties in emotion regulation is, moreover, delineated by the results of a Canadian study [5] postulating the unique contribution of emotional abuse and dysregulation of emotions in the development of BPD.

Various studies have evidently demonstrated the association between Borderline Personality Disorder, difficulties in emotion regulation as well as sexual, physical and emotional abuse, which increase is favored by multiple factors including, but not limited to the victim’s gender and age, the family’s structure and size, the intra-familial communication and the parents’ level of education [16]. Nevertheless, reflection on the relationship between BPD and a unique form of abuse, specifically emotional abuse remains indistinct within the national literature.

On another note, the Lebanese population is moving with a stagger through the country’s innumerable crises, be it the political and/or financial turmoil, the August 2020 Beirut Port explosions’ aftermath or the COVID-19 pandemic [53]. As a result of the society’s malfunctioning, children are being deprived of their basic needs and rights which primarily include health, education, housing, food, and protection. Consequently, childhood abuse is on the rise; “one in two children in Lebanon is at serious risk of physical, emotional, or sexual violence” [54]. In fact, aiming to gain a certain amount of money by receiving dowries or make ends meet, parents are resorting to inadequate, nay harmful coping strategies such as forcing their children into abuses namely child marriage or labor [53]. Particularly relating to emotional abuse, humiliation, insults, and threats of abandonment have been employed by parents from the Arab World towards their children, raising the prevalence of this form of abuse to over 50% according to some studies [16, 55–57].

To remedy such matters, one would turn towards child protection law and system that is nationally defined by statutory measures under Law 422 on “Protection of Minors in Conflict with the Law or At Risk”. Nevertheless, the latter, which has last been revised 16 years ago, is neither applied nor applicable for victims and is limited to outrageous cases of abuse. Consistently, Law 422 is neither preventive nor protective; it functions as a last resort measure [58]. The urgency of this situation and the significant and long-lasting implications these instances of childhood abuse—specifically that emotional—might have, including but not limited to detrimental impacts on one’s mental wellbeing, increased affect dysregulation, and personality disorders [59] directed the choice of exploring this form of abuse and selecting it as one of the study’s variables.

Additional repercussions to the myriad upheavals experienced by the Lebanese population through the course of the years reveal numerous mental health problems leading to difficulties in emotion regulation [60]. In fact, mental health challenges such as depression and anxiety influence emotion regulation abilities, understanding one’s emotions, and managing their negative effects [61–63]. Similarly, difficulties in emotion regulation have been consistently linked to various adverse psychological and behavioral outcomes, such as BPD [64]. Given that research on emotion regulation lack in Lebanon [60], verifying whether the aforementioned specifics and its link to challenges in regulating one’s emotions among the country’s citizens was deemed to be significant.

Moreover, public stigma and misconceptions against mental illness are still detectable among the Lebanese population [65, 66]. Until the present day, individuals of Lebanese nationality deny the existence and occurrence of mental illness or avoid seeking help from mental health professionals in fear of peers’ reactions [65].

Considering the entirety of the data reported above, the present research aimed at examining the mediating role of difficulties in emotion regulation in the relationship between childhood emotional abuse and Borderline Personality Disorder traits among the Lebanese population given its absence in the national literature and the importance of exploring susceptibility factors of BPD. On that account, the formulated hypothesis for this study suggests that difficulties in emotion regulation have a mediating role in the association between childhood emotional abuse and BPD traits among Lebanese adults. More specifically, victims of this type of abuse among the said population might be associated with the manifestation of BPD traits during adulthood, mediated by high levels of difficulties in emotion regulation.

## Methods

### Study design and procedure

This cross-sectional study was conducted between the months of March and August 2022, among the adult Lebanese population residing in all of Lebanon’s governorates (Beirut, Mount Lebanon, North, South and Bekaa). The “Snowball Sampling” technique using Google Forms was carried out to collect the necessary data for the investigation. Indeed, participants were first invited to complete the questionnaire which link was initially distributed via social media applications such as ‘WhatsApp’, ‘Instagram’ and ‘Facebook’, and then asked to share it with their acquaintances, friends and/or family members. First recruited participants comprise acquaintances of the present researchers, to whom they are connected on their social media accounts. In other words, the former would have visited the Google Forms link shared on the said online platforms, originally on the researchers’ profile, then completed the study’s questionnaire and followed with the Snowball Sampling method for further data collection.

Prior to proceeding with the data collection, a pilot study composed of 15 members was initiated with the objective of evaluating the duration and feasibility of the questionnaire as well as correcting potential errors and misunderstandings related to its items.

### Participants

The study’s sample consisted of 411 Lebanese adults aged between 18 and 65. Excluded were participants outside this age range, those who are not of Lebanese nationality and those who did not wish to contribute to this research project. Adults over the age of 65 were indeed excluded in order to eliminate the risk of any potential mental impairment’s presence [67]. The latter could possibly be due to dementia which generally begins during the second half of one’s life [68] and whose confirmation from the participant’s side cannot be satisfied by a self-administered questionnaire. Subjects with a diagnosis of Schizophrenia, Bipolar Disorder or Attention Deficit Hyperactivity Disorder previously made by a psychiatrist or a specialized physician, were also excluded from the study, as well as those taking psychotropic medications, as it could affect their cognitions and their ability to understand or fill the questionnaire [42]. The entirety of the above-cited information was obtained through participants’ responses on the self-administered questionnaire.

Before proceeding with the questionnaire, participants were informed of the purpose of the study, assured of the anonymity of their participation and provided with a virtual informed consent form via ‘Google Forms’. The latter had to be ‘signed’, after reading, by clicking the box ‘Yes, I acknowledge having read the above-mentioned information and I agree to participate in this study voluntarily and without any pressure’ to which the answer is required in order to continue with the self-administration. Participants had the right to accept or refuse to respond and no financial compensation was provided in exchange for their submission.

### Sample size calculation

Using the below formula suggested by Fritz and MacKinnon [69] to estimate the sample size required to detect a mediated effect, a minimal sample of 126 participants was deemed necessary: $n=\frac{L}{{f}^{2}}+k+1$ where $f$ = 0.26 for moderate effect size, $L$ = 7.85 for an α error of 5% and power β = 80%, and $k$ = 9 variables to be entered in the model.

### Questionnaire and measures

The self-administered questionnaire used to collect essential information for the study, was developed in Arabic, Lebanon’s official language, and required approximately 15 min to complete. The first part of the questionnaire evaluated the participant’s socio-demographic data: nationality, governorate of residence, age, gender, marital status, level of education and household crowding index. The latter reflects the socio-economic status of the family and was calculated by dividing the number of people residing in the house by the number of rooms that form it, apart from the bathrooms and the kitchen [70]. Additional information was collected through the participants’ responses to assess the exclusion criteria set for the study and cited hereinabove.

The second part of the questionnaire consisted of the following measures:

#### Difficulties in emotion regulation scale—brief version (DERS-16)

The DERS-16 [71] is a self-report questionnaire that assesses individuals’ difficulties in regulating emotions through the following dimensions: non-acceptance of negative emotions, limited access to emotion regulation strategies perceived as effective, difficulties in controlling impulsive behavior when experiencing negative emotions, inability to adopt goal-oriented behaviors when in distress and lack of emotional awareness and clarity. Participants are to rate 16 items such as “*I have difficulty making sense out of my feelings”*, on a 5-point Likert scale ranging from ‘*almost never*’ to ‘*almost always*’. The higher the scores, the greater the difficulty in emotion regulation. Additionally, this scale demonstrates remarkable psychometric properties both in clinical and non-clinical groups [71]. More specifically, the DERS-16 returns excellent internal consistency (Cronbach’s α = .92), adequate reliability in test-retest (ρI = .85; p < .001), equivalent construct validity demonstrated through correlations with the original 36-item DERS, as well as satisfactory discriminant validity indicated by significant correlations of its scores with measures of negative emotionality, psychiatric symptoms, experiential avoidance, mindfulness, BPD manifestations, deliberate self-harm, and alcohol use disorder [71]. The Arabic version of the DERS-16 was used in this study [72] (Cronbach’s alpha = .95).

#### Childhood trauma questionnaire—short form (CTQ-SF)

The CTQ-SF [73] is a retrospective self-report questionnaire, which confers a subjective assessment of a participant’s general childhood environment along five domains: emotional abuse “*I felt that someone in my family hated me*”, emotional neglect “*I felt loved*”, physical abuse “*people in my family hit me so hard that it left me with bruises or marks*”, physical neglect “*I had to wear dirty clothes*” and sexual abuse “*someone molested me*”. Participants rate 28 items on a 5-point Likert scale ranging from ‘never true’ to ‘very often true’. The scores reveal the degree of abuse; the higher the score, the greater the severity of childhood abuse and/or neglect. On another note, internal consistency among the subscales of the CTQ-SF is reasonably strong; in a community sample, alpha coefficients equal to 0.87 for emotional abuse, 0.83 for physical abuse, 0.92 for sexual abuse, 0.91 for emotional neglect, 0.61 for physical neglect and 0.70 for the total score [73]. The Arabic translation of the CTQ-SF, used in previous studies [74] was utilized in this research.

#### Borderline personality questionnaire (BPQ)

The BPQ [75] is a self-report questionnaire that assesses Borderline Personality Disorder traits, which are shown to be significantly convergent with the said disorder. Participants respond to 80 dichotomous items such as “*I often do things without thinking them through*”. The presence of Borderline Personality Disorder is assessed according to the scores obtained; a standard deviation of 1.5 above the mean is considered clinically significant. The BPQ reveals high internal consistency overall and reliability ranging from reasonable to strong for most subscales given its length. Moreover, results indicate good discriminant validity and significant convergent validity [75]. The Arabic version of the BPQ was used in this study [76] (Cronbach’s alpha = 0.94).

### Data analysis

The SPSS software v.25 was used to complete the statistical analysis. The score of the “Borderline Personality Questionnaire (BPQ)” [75] was estimated as normally distributed given that the values of skewness (= 0.604) and kurtosis (=-0.376) fluctuated between − 1 and + 1 [77]. In addition, Student t test was used to compare two means and the Pearson test to correlate two continuous variables. A linear regression was performed, using the ENTER method, while considering the BPQ score as the dependent variable.

The mediation analysis was conducted using PROCESS SPSS Macro v. 3.4, model 4’ [78]; three pathways were calculated: (a) relationship between childhood emotional abuse and difficulties in emotion regulation; (b) relationship between difficulties in emotion regulation and Borderline Personality Disorder; (c’) direct effect of the relationship between childhood emotional abuse and Borderline Personality Disorder; and (c) total effect of the relationship between childhood emotional abuse and Borderline Personality Disorder. Finally, the results of the linear and mediation models were adjusted for all the variables that showed a *p* < .25 in the bivariate analysis. *P* < .05 was deemed statistically significant.

## Results

### Sociodemographic and other characteristics of the sample

Four hundred eleven participants participated in this study, with a mean age of 32.86 ± 11.98 years and 75.4% females. Other descriptive statistics of the sample can be found in Table 1.

**Table 1:** Sociodemographic and other characteristics of the sample (N = 411)

Variable	N (%)
Sex	
Male	101 (24.6%)
Female	310 (75.4%)
Marital status	
Single	254 (61.8%)
Married	157 (38.2%)
Education level	
Secondary or less	39 (9.5%)
University	372 (90.5%)
Governorate	
Beirut	40 (9.7%)
Mount Lebanon	295 (71.8%)
North	26 (6.3%)
South	27 (6.6%)
Bekaa	23 (5.6%)
	**Mean ± SD**
Age (years)	32.86 ± 11.98
Household crowding index (persons/room)	0.93 ± 0.44
BPQ score	24.78 ± 14.22
DERS-16 score	34.00 ± 13.02
CTQ-SF emotional abuse	8.54 ± 4.26
CTQ-SF physical abuse	6.73 ± 3.09
CTQ-SF sexual abuse	9.94 ± 2.16
CTQ-SF emotional neglect	13.70 ± 3.94
CTQ-SF physical neglect	8.22 ± 2.77
CTQ-SF minimization denial	9.31 ± 3.12

### Bivariate analysis of factors associated with borderline personality disorder

The results of the bivariate analysis of factors associated with Borderline Personality Disorder are summarized in Tables 2 and 3. The results showed that single participants had higher BPQ scores than married ones. In addition, higher BPQ scores were significantly associated with higher scores on DERS-16, emotional, physical, and sexual abuse, emotional and physical neglect, whereas higher minimization/denial scores and older age were significantly associated with lower BPQ scores.

**Table 2:** Bivariate analysis of factors associated with Borderline Personality Disorder

Variable	Borderline Personality Disorder (mean ± SD)	*p*
Sex		0.656
Male	24.23 ± 13.73	
Female	24.95 ± 14.40	
Marital status		**< 0.001**
Single	27.58 ± 14.33	
Married	20.24 ± 12.86	
Education level		0.630
Secondary or less	25.82 ± 12.82	
University	24.67 ± 14.37	

Numbers in bold indicate significant *p*-values

**Table 3:** Correlations of continuous variables with Borderline Personality Disorder

	1	2	3	4	5	6	7	8	9	10
1. BPQ	1									
2. DERS-16	0.61***	1								
3. CTQ-SF emotional abuse	0.42***	0.43***	1							
4. CTQ-SF physical abuse	0.24***	0.23***	0.65***	1						
5. CTQ-SF sexual abuse	0.22***	0.23***	0.45***	0.64***	1					
6. CTQ-SF emotional neglect	0.27***	0.09	0.27***	0.20***	0.06	1				
7. CTQ-SF physical neglect	0.25***	0.08	0.28***	0.34***	0.28***	0.52**	1			
8. CTQ-SF minimization/denial	− 0.27***	− 0.15**	− 0.39***	− 0.31***	− 0.18***	− 0.63***	− 0.38***	1		
9. Age	− 0.22***	− 0.34***	− 0.24***	− 0.08	− 0.05	− 0.001	0.07	− 0.04	1	
10. Household crowding index	− 0.01	0.03	− 0.002	0.03	0.02	0.03	0.03	0.03	− 0.10*	1

*p < .05; **p < .01; ***p < .001; BPQ = Borderline Personality Questionnaire; CTQ-SF = Childhood Trauma Questionnaire—Short Form; DERS-16 = Difficulties in Emotion Regulation Scale—Brief Version

### Multivariable analysis

The results of a linear regression (ENTER method), taking the Borderline Personality Questionnaire score as the dependent variable, showed that higher scores on DERS-16 (Beta = 0.58), emotional abuse (Beta = 0.41), emotional neglect (Beta = 0.41) and physical neglect (Beta = 0.51) were significantly associated with higher BPQ scores (Table 4).

**Table 4:** Multivariable analysis: Linear regression (ENTER method) taking the Borderline Personality Questionnaire score as the dependent variable

Variable	Unstandardized Beta	Standardized Beta	*p*	95% CI
DERS-16	0.58	0.53	**< 0.001**	0.49; 0.67
CTQ-SF emotional abuse	0.41	0.12	**0.026**	0.05; 0.77
CTQ-SF physical abuse	− 0.17	− 0.04	0.522	− 0.69; 0.35
CTQ-SF sexual abuse	0.07	0.01	0.828	− 0.57; 0.71
CTQ-SF emotional neglect	0.41	0.11	**0.032**	0.04; 0.79
CTQ-SF physical neglect	0.51	0.10	**0.034**	0.04; 0.97
CTQ-SF minimization/denial	− 0.18	− 0.04	0.423	− 0.64; 0.27
Age	0.02	0.02	0.716	− 0.09; 0.14
Marital status (married vs. single*)	-2.09	− 0.07	0.129	-4.80; 0.61

Numbers in bold indicate significant *p***-**values; Nagelkerke R^2^ = 0.45; CTQ-SF = Childhood Trauma Questionnaire—Short Form; DERS-16 = Difficulties in Emotion Regulation Scale—Brief Version

### Indirect effect analysis

**Fig. 1:**
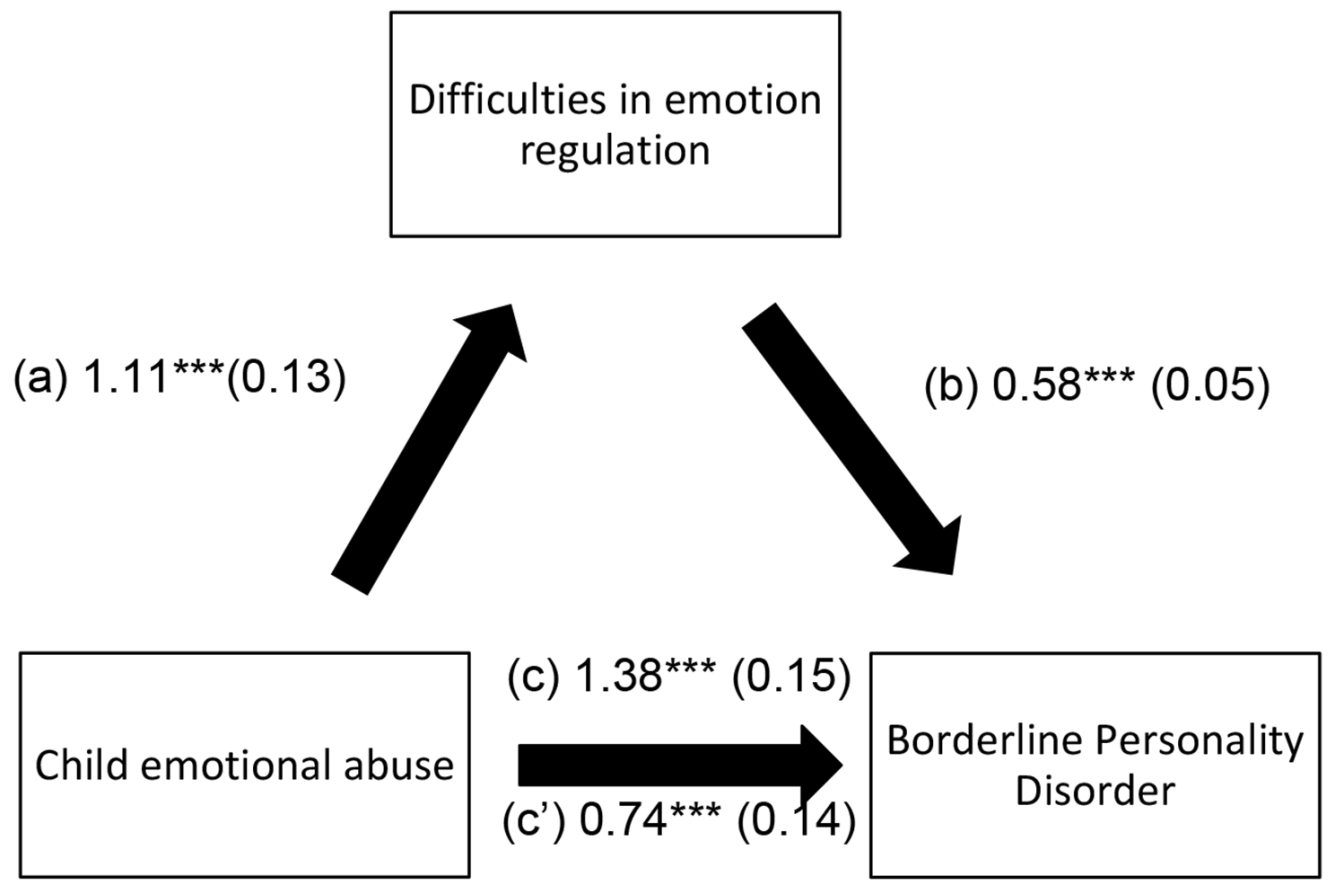
(**a**) Relation between child emotional abuse and difficulties in emotion regulation (R^2^ = 0.254); (**b**) Relation between difficulties in emotion regulation and Borderline Personality Disorder (R^2^ = 0.435); (**c**) Total effect of the relation between child emotional abuse and Borderline Personality Disorder (R^2^ = 0.225); (c’) Direct effect of the relation between child emotional abuse and Borderline Personality Disorder. Numbers are displayed as regression coefficients (standard error). ***p < .001

The results of the indirect effect analysis are shown in Table 5, with detailed results summarized in Fig. 1. Difficulties in emotion regulation played an indirect effect role between child emotional abuse and Borderline Personality Disorder. Higher child emotional abuse scores were significantly associated with higher DERS-16 scores, which in turn were significantly associated with higher BPQ scores. Moreover, childhood emotional abuse scores were directly associated with higher BPQ scores.

**Table 5:** Indirect effect analyses results, taking CTQ-SF emotional abuse subscale as the independent variable, difficulties in emotion regulation as the mediator and Borderline Personality Disorder as the dependent variable

Independent variable	Direct effect			Indirect effect		
	**Beta**	**SE**	***P***	**Beta**	**Boot SE**	**Boot CI**
CTQ-SF emotional abuse	0.74	0.14	< 0.001	0.64	0.10	0.45; 0.86*

* indicates significant indirect effect

## Discussion

The results of the present research are in line with several previously conducted studies [5, 79]; they revealed the potential existence of an indirect effect of the DERS-16 in the relationship between childhood emotional abuse and Borderline Personality Disorder, confirming the hypothesis formulated in this paper. Additionally, the study results impart supplementary specificity to those of van Dijke et al. [42], insofar as their findings reveal partial mediation of affect downregulation in the relationship between BPD manifestations and trauma experienced during childhood. These results, implicating disturbed regulation of emotions and childhood experiences of trauma, may act as a basis for the identification of individuals with Borderline Personality Disorder [42]. For instance, chronic reactions to traumatic events and subsequent difficulties with emotion regulation may be the main cause for BPD among some subjects, while among others, the latter may not be the primary factor. This distinction can aid in the development of personalized treatment plans for adults with BPD and guide clinical and scientific testing along treatment methods depending on the role of emotion dysregulation and childhood adversity [42].

On a further note, through his biosocial model, considered influential on the etiology of BPD, Linehan [32] hypothesized that the manifestations of Borderline Personality Disorder are the reflection of difficulties in emotion regulation. In fact, these difficulties seem to stem from the relationship between an early disabling environment and a preexisting emotional vulnerability [15]. The latter could be described as a biological predisposition to an intense and unstable negative affect characterized by heightened sensitivity to emotions, followed by a gradual return to a baseline emotional state [15].

### Emotional abuse and borderline personality disorder

Although various forms of abuse inflicted during childhood translate the essence of disrupted social environments in multiple developmental models [6, 32, 80], that of emotional abuse highlights a particular association with Borderline Personality Disorder in accordance with the results of the present study. The latter are consistent with the results of previous research considering the disabling and unstable environment of the child, particularly that characterized by emotional abuse, as the basis for BPD development [14, 15, 32, 81, 82].

As a matter of fact, individuals who experience emotional abuse in their upbringing may develop negative perceptions of themselves and struggle to respond to changing environments [83, 84]. Such type of abuse could indeed directly convey a detrimental self-image and instill beliefs that one is unwanted, worthless, or flawed [83, 84]. In other words, experiencing emotional abuse can leave individuals feeling invalidated, insecure, and uncertain about their thoughts and emotions [85]. The aforementioned can lead to difficulties in regulating one’s emotions and self-image, which, in turn, are significant features of BPD [86]. Along those lines, and consistent with previous research, an emotionally abusive rearing environment may subsequently hinder healthy personality development and potentially be more harmful than physical or sexual abuse and thus, increase the likelihood of Borderline Personality Disorder [83, 84, 87, 88].

In addition, the substantial effects found for emotional abuse align with previous studies and theories linking them to emotional invalidation and sensitivity to rejection [89–91]. In fact, victims of early emotional abuse may become highly attuned to interpersonal cues such as facial expressions and tone of voice, which can make them more likely to perceive rejection and abandonment in social settings [92]. This heightened sensitivity may contribute to the fear of abandonment and unstable relationships that are common traits of BPD [92]. Emotional abuse may also contribute to the development of maladaptive coping mechanisms, such as self-harm or substance abuse which are frequently linked to BPD [93]. This often is a result of attempting to manage the intense emotions and distress brought on by this type of abuse [93].

While emotional abuse is one of many potential risk factors for BPD, not everyone who experiences it will develop the disorder [94]. Similarly, not every individual with BPD has suffered early emotional abuse, and there are many other factors that can contribute to the development of the disorder, some of which are genetics, childhood experiences, and other environmental factors [95].

### Clinical implications

Along with the findings relating that difficulties in emotion regulation mediate the relationship between BPD and childhood emotional abuse, training sessions on various strategies including those on emotion regulation could potentially benefit victims of early incidents of emotional abuse. These comprise (1) cognitive reappraisal which involves reassessing how one initially perceives a situation and altering its emotional significance in order to modify the emotional reaction towards that specific situation, as well as (2) expressive suppression which, in contrast, implicates the act of inhibiting the behavioral manifestation of an emotion with the aim of reducing the emotional impact of events [60]. In fact, these strategies would serve as preliminary to therapeutic interventions focused on cognition and would allow the establishment of a sense of security and reliance among individuals victim of childhood emotional abuse [96] and concurrently manifesting BPD. Subsequently, the latter would be the precise audience of a wide range of therapeutic interventions including dialectical behavior therapy [97], trauma-focused cognitive behavioral therapy, or Eye Movement Desensitization and Reprocessing. Indeed, once the feeling of security has been established, these interventions would in turn promote and reinforce emotion regulation [98] as well as distress modulation and formation of healthy relationships [99].

Numerous other tactics may be taught and implemented by communities to work towards creating a safe and supportive environment for children and families, and, in other words, help prevent child abuse and promote healthy emotional development [100–102]. Some of these involve building strong community connections; by creating an unyielding network of individuals, families, and organizations, communities can work together to identify and address the underlying causes of child abuse and can provide support, resources, and services that may aid in preventing its occurrence. Encouraging positive parenting could also be an effective tool; using positive reinforcement, setting clear boundaries, and modeling appropriate behavior, can improve a child’s emotional regulation capacities and reduce the risk of abuse. In that context, communities may support parents by providing access to resources and parenting programs that target positive parenting strategies. Another technique would be to educate community members on the signs of child abuse and increase their awareness and understanding so individuals are better equipped to identify and intervene in situations where a child may be at risk. Promoting mental health awareness and providing access to mental health and/or crisis intervention services, and support groups is of even significance to the aforementioned points, given that difficulties on the mental health level risk of contributing to both child abuse and poor emotion regulation strategies. On that account, and considering that Lebanese people often refuse to acknowledge the incidence of mental health challenges and avoid to seek assistance from mental health practitioners due to apprehension about how their peers may react [65], fostering a culture of support where individuals feel comfortable seeking help when needed can be key in preventing child abuse and improving one’s emotion regulation competences as well as their overall wellbeing whether suffering from a personality disorder such as BPD.

### Limitations

The sample recruited was nonclinical but part of the general population, which would blur the accuracy of the associations identified among individuals with BPD, and reported in the analyzed results. Replicating the research among a clinical sample would thus have merit. Additionally, being a cross-sectional study, the variability over time could not be appreciated. Consequently, in order to expeditiously attest to the links associating childhood emotional abuse, Borderline Personality Disorder and difficulties in emotion regulation, longitudinal monitoring of the participants would prove to be essential. Moreover, due to having employed a cross-sectional design for the study, drawing inferences around causation link has been precluded. The utilization of bot detection to exclude any possible invalid response collected through the Snowball Sampling technique was not made. The exclusive use of retrospective self-assessment screening tools would constitute an additional limitation to the study. For instance, BPD diagnoses were solely verified through the questionnaire completed online and thus, without clinical examination. Also, early experiences of emotional abuse may be subject to recall biases and would likely be distorted or misunderstood by people with BPD among whom accurate and coherent representation of their own individuality and that of others presents a lesser subtlety. Hence, in order to overcome these limitations, the utilization in future studies of a wider and more varied range of screening tools—particularly clinical interviews for the assessment of Borderline Personality Disorder—validated in Lebanon, would be suggested. Furthermore, this study may have run the risk of collecting intentional falsehoods in the participants’ responses, led by a fear of revealing themselves to science and/or exposing themselves to strangers, although confidentiality and anonymity of their contribution was assured.

## Conclusion

The results of this study revealed that individuals who were emotionally abused during their childhood tend to present Borderline Personality Disorder, mediated by a high level of difficulties in emotion regulation. In Lebanon, the number of individuals exposed to early experiences of abuse is far from reassuring [103], pointing to the need of implementing policies and interventions aiming to approach this matter. An enhanced knowledge by future studies of the specificity of a unique form of childhood abuse/neglect as a precursor of BPD would be crucial, given that the latter is also predicted by a plurality of genes/environment interactions [8], and that a multitude of combinations of forms of abuse subsist. Considering the whole family dynamic’s responsibility in the prediction of this personality disorder would be interesting, taking into account parenting practices, parent/child interaction and relationship, or even parental psychopathology. Lastly, replicating the present study among a clinical sample would have merit and would explicate the validity of the results obtained from individuals diagnosed with Borderline Personality Disorder.

## Data Availability

All data generated or analyzed during this study are not publicly available to maintain the privacy of the individuals’ identities. The dataset supporting the conclusions is available upon request to the corresponding author.
